# Distributed Deep Fusion Predictor for a Multi-Sensor System Based on Causality Entropy

**DOI:** 10.3390/e23020219

**Published:** 2021-02-11

**Authors:** Xue-Bo Jin, Xing-Hong Yu, Ting-Li Su, Dan-Ni Yang, Yu-Ting Bai, Jian-Lei Kong, Li Wang

**Affiliations:** 1 Artificial Intelligence College, Beijing Technology and Business University, Beijing 10048, China; yuxinghong@st.btbu.edu.cn (X.-H.Y.); baiyuting@btbu.edu.cn (Y.-T.B.); kongjianlei@btbu.edu.cn (J.-L.K.); 2 China Light Industry Key Laboratory of Industrial Internet and Big Data Beijing Technology and Business University, Beijing 10048, China; 3 Electrical and Information Engineering College, Tianjin University, Tianjin 300072, China; 18693803229@163.com

**Keywords:** series causality analysis, Bayesian LSTM, multi-sensor system, meteorological data, big measurement data, deep fusion predictor

## Abstract

Trend prediction based on sensor data in a multi-sensor system is an important topic. As the number of sensors increases, we can measure and store more and more data. However, the increase in data has not effectively improved prediction performance. This paper focuses on this problem and presents a distributed predictor that can overcome unrelated data and sensor noise: First, we define the causality entropy to calculate the measurement’s causality. Then, the series causality coefficient (SCC) is proposed to select the high causal measurement as the input data. To overcome the traditional deep learning network’s over-fitting to the sensor noise, the Bayesian method is used to obtain the weight distribution characteristics of the sub-predictor network. A multi-layer perceptron (MLP) is constructed as the fusion layer to fuse the results from different sub-predictors. The experiments were implemented to verify the effectiveness of the proposed method by meteorological data from Beijing. The results show that the proposed predictor can effectively model the multi-sensor system’s big measurement data to improve prediction performance.

## 1. Introduction

Measurements have been obtained and saved in many multi-sensor systems, such as mobile robots [1], unmanned aerial vehicles (UAVs) [2,3], smart agriculture [4,5], air quality monitoring systems [6,7], etc. It is very meaningful to analyze these data and understand and predict the information in the sensor system [8], for example the analysis and prediction of meteorological elements in precision agriculture or environmental management systems [9]. Furthermore, in terms of environmental governance, the prediction for air pollution sources such as PM2.5 has played an important role [10,11,12,13].

Recently, more measurements have been collected with the development of sensor technology. Therefore, in a multi-sensor system, big data analysis has become a new research area. These data have two characteristics: noisy and numerous [14]. For example, the collected and saved meteorological data are big data and include many variables, such as temperature, wind, rainfall, humidity, etc. Further, they are related to each other [15]. However, the correlation between each type of variable is different: some of them have a strong correlation, but some have a low correlation.

In general, more data can provide more information. For big data, deep learning can extract hidden information to make more accurate predictions [16]. The recent research has proven that the recurrent neural network (RNN) and its improved version are widely used in regression prediction problems with better nonlinear modeling ability compared with the classical regression method.

We can find that the network has become larger and more complex due to the massive amount of data. However, because of the amount of data, the network’s training time is getting longer. To make matters worse, the increase of the input data does not improve the prediction performance; on the contrary, it decreases.

This paper focuses on how to use this big noisy data in the multi-sensor system to efficiently improve prediction performance. This paper mainly aims at multi-sensor systems, proposes a causal entropy method for feature selection, and constructs a distributed forward multi-step prediction framework based on Bayesian deep learning theory. In this way, the dimensionality reduction of high-dimensional data feature selection is realized, and the problem of data noise affecting deep network training is initially overcome. The rest is organized as follows: Section 2 summarizes current prediction models and describes the main contribution of this paper. Section 3 proposes a distributed deep learning network predictor, and Section 4 describes the experiments and results to verify the performance of our predictor. We draw conclusions in Section 5.

## 2. Related Works

### 2.1. The Methods for Prediction

Prediction is to analyze historical data and obtain the trend of the future. With the development of computer storage technology and sensor technology, the prediction of measurement data in a multi-sensor system has been widely used in many fields. It has become a hot topic of research. The traditional prediction methods require prior knowledge of the data, such as exponential smoothing [17], moving average (MA) [18], auto-regression (AR) [19], auto-regressive integrated moving average (ARIMA) [20], etc. In practical systems, the traditional prediction methods cannot obtain a high accuracy prediction result due to the system’s complexity.

For nonlinear input data, shallow machine learning methods obtain model parameters through training, such as support vector machines (SVMs) [21], the echo state network (ESO) [22], Boltzmann machines (BMs) [23], shallow artificial neural networks (ANNs) [24], generalized regression neural networks (GRNNs) [25], etc., which avoids the requirement of prior knowledge of the data. However, because of their simple structure, they cannot process large amounts of data.

With the development of the depth of the neural network, the hidden information in the massive data can be extracted to make more accurate predictions. The recurrent neural network (RNN) [26] and its improved versions, such as long short-term memory (LSTM) [27], etc., is widely used for regression prediction problems, demonstrating its superior nonlinear modeling capabilities. For example, a gated recurrent unit (GRU) network [28] and Bi-LSTM [29] were proposed to improve LSTM. Furthermore, researchers [30,31] have combined the one-dimensional convolutional neural network (CNN) with LSTM to predict the time series data. System identification is the theory, and the methods of establishing the mathematical models of dynamical systems [32,33,34,35,36] and some identification approaches can be used to establish the prediction models and soft sensor models [37,38,39,40,41,42] for various application problems.

### 2.2. The Method to Calculate Causality and Correlation

Undoubtedly, deep neural networks are currently the best solution to the big data prediction problem of multi-sensor systems. However, we found that the network’s ability to predict does not increase as the amount of input variable increases. On the contrary, sometimes, the larger the amount of input measurement data from multi-sensor system, the worse performance the prediction obtains. This is contrary to what we have always believed: one advantage of the deep learning network is that it has comprehensive and robust learning capabilities for big data.

We believe one of the reasons is that the data contain too much low-relevance information; the increase in the amount of data leads to a decrease in the ratio of useful information. The weights of training for the neural network and the diluted information make the network’s convergence more difficult, so the prediction performance cannot be developed, but is even reduced. Therefore, we think the data with a high correlation and strong causality with the target variable should be selected as the network’s input data, rather than just increasing the number.

Then, we describe the correlation degree method and discuss a causal correlation method for measuring variables suitable for multi-sensor systems to measure big data. The Pearson correlation coefficient (PCC) [43] and Spearman correlation coefficient [44,45] have been used for such a problem. The former can be used to find the linear relationship between the two variables. For the data, the features are continuous and conform to a positive distribution; the linear relationship between the two variables can be mined by the PCC [46]. Jing et al. [47] selected the characteristic sub-sequence by PCC to improve the prediction accuracy when forecasting photo-voltaic power output. Lin et al. [48] built a hybrid model framework using the stacking scheme of integrated learning by PCC between different models. As for the prediction problem, PCC requires a known prediction target variable, so it cannot be applied for predicting.

Spearman’s correlation coefficient is mainly used to solve problems related to sequential data. It applies to two variables with an order relationship. Another kind of correlation analysis method is called the Kendall correlation coefficient [49], which is suitable for sequenced variables or evenly spaced data that do not satisfy the assumption of a normal distribution. This method is usually calculated for a piece of sequence data, and it cannot obtain an effective correlation between the input and output for large amounts of data.

Contreras-Reyes et al. [50] used the frequency-domain Granger-causality method to test the statistical significance of causality between two time series and determine the direction of causality on the drivers of pelagic species’ biological indicators. Since the Granger causality coefficient is used in two stationary time series, its application is limited. Podobnik et al. [51] proposed a detrending cross-correlation analysis method to explore the correlation between two non-stationary series. It shows that effectively measuring the correlation between two variables can help analyze the change characteristics of one of the variables.

The current methods to calculate the correlation and causality rely on the predicted result and cannot be applied to the prediction problem of multi-sensor systems.

### 2.3. The Bayesian Deep Learning Network

The big data measured by sensors contain noise, which is another reason for the degradation of prediction performance based on deep learning networks. Traditional neural network training obtains the fixed weights and biases, which are easily disturbed by noise [52,53]. On the one hand, the noise makes it difficult to converge the network, that is the loss of the network is larger. On the contrary, if the noise is also learned as a certain value until a small loss value is obtained, it will cause the problem of overfitting [54].

Suppose we use the data distribution to train the network and obtain weights and deviations to express the input data’s distribution characteristics. In that case, the problem of overfitting will be avoided. Based on the distribution characteristics of the weights and deviations, the obtained neural network is a group. The output is also a group of prediction outputs with distribution characteristics, improving the prediction results’ reliability. Based on this research question, the Bayesian deep learning network came into being [55]. Through Monte Carlo sampling, the Bayesian deep learning network trains the network several times and takes the average of all losses, then uses it for backpropagation to obtain the distribution of weights and deviations [56].

The Bayesian method has been used in many application systems, such as indoor tracking [57], robot systems [58], etc. The Bayesian deep learning network has been applied in modeling with noisy data, and some results have been obtained. For example, Li et al. [59] integrated uncertainties by defining the Bayesian deep learning framework, in which a sequential Bayesian boosting algorithm is used to improve the estimation accuracy. Another example is [60], where a Bayesian framework was proposed to model the valence predictions.

### 2.4. Innovation

Aiming at the problem of improving the prediction performance based on the huge amount measurement data in a multi-sensor system, this paper provides a distributed deep prediction network. To solve the contradiction between data volume and performance and the influence of data noise on prediction performance, the innovation of this paper lies in the following:

(1) A series causality entropy method is developed to select the related input data for the neural network. Compared with the PCC [48], Spearman correlation [45], and the Kendall correlation coefficient method [61], the method does not depend on the prediction results and is suitable for prediction problems based on measurement data in multi-sensor systems.

(2) A distributed prediction framework is proposed, in which Bayesian training is used to suppress the noise impact of the data, and the prediction based on the selected input data is fused by a nonlinear fusion network. Compared with the classical LSTM [27], GRU [28], CNN-LSTM [11], the conv-LSTM [30] predictor, etc., the proposed method outperforms them in its prediction performance.

## 3. Distributed Deep Fusion Predictor

### 3.1. Series Causality Entropy

**Figure 1:**
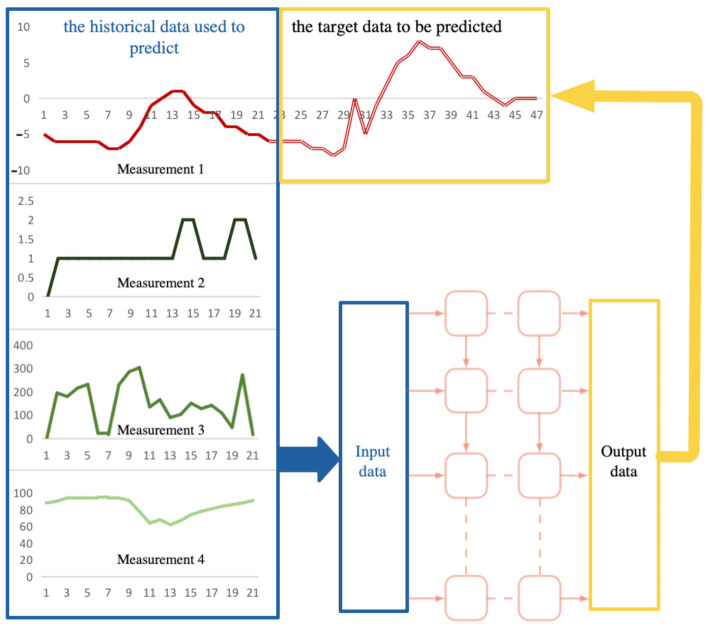
Relationship between the target variables to be predicted and the input variables.

In a multi-sensor system, we can set up multiple sensors to obtain a variety of measurement data. For example, in the system given in Figure 1, we use four sensors to obtain four types of measurement data, and they will be used as candidate input data for the deep network. The prediction task is for Measurement 1, and we will predict its future trend.

Firstly, we will consider the method to select the input data for the networks. Obviously, the principle of selecting data is to select those measurement data that are most causal for the future trend of Measurement 1. As for the prediction problem, it can be defined as the series causality between the historical data and the future data.

We give the following definition about the causality entropy to calculate the measurement’s causality between two data named *X* and *Y*:

(1) $$\begin{array}{c} \begin{array}{c} CE\left(X,Y\right)=\frac{1}{N-1}\sum_{i=1}^{N}\left(\frac{X\left(i\right)-\bar{X}}{\sigma_{X}}\right)\times \log\left(\frac{Y\left(i\right)-\bar{Y}}{\sigma_{Y}}\right) \end{array} \end{array}$$

where $\bar{X}$ and $\bar{Y}$ are the mean of X(i) and Y(i), i=1,2,……,N, respectively, and $\sigma_{X}$ and $\sigma_{Y}$ are the standard deviation of X(i) and Y(i). We can see that CE(X,Y) can be positive or negative. When it is a positive number, it indicates that the two data are positively supporting. Otherwise, it is negatively supporting.

This calculating correlation method cannot be directly applied to obtain the correlation of prediction problems. Because the measured data and their prediction are considered in the prediction problem, therefore, we cannot calculate CE when the prediction is not yet available. Secondly, since the step length of the measurement data *I* is different from the predicted step length *J*, the number of data points *N* in Equation (1) cannot be used.

Therefore, we propose the following series causality coefficient (SCC) for the measurement in the multi-sensor system. Suppose the measured data are represented by $X_{m}\left(i\right)$, where m=1,2,……,M is the sensor number to obtain the measurement and i=1,2,……,I is the step number of historical data used for prediction. The target data to be predicted are represented by Y(j), where j=1,2,……,J is the step number of prediction. We revised the method to calculate coefficient Equation (1) as the following.

(2) $$\begin{array}{c} \begin{array}{c} S_{m}=\frac{1}{K-1}\sum_{i=1,j=1}^{K}\left|\frac{X_{m}\left(i\right)-\bar{X_{m}}}{\sigma_{X_{m}}}\right|\times \log\left|\frac{Y\left(j\right)-\bar{Y}}{\sigma_{Y}}\right| \end{array} \end{array}$$

where m=1,2,……M is the sensor number to obtain the measurement, K=min(I,J), $\bar{X_{n}}$ and $\bar{Y}$ are the mean of Xm(i) and Y(i), i=1,2,……K, respectively, and $\sigma_{X_{m}}$ and $\sigma_{Y}$ are the standard deviation of $\bar{X_{n}}$ and $\bar{Y}$. We can find that Equation (2) still has the prediction Y(i), which is unknown data. To eliminate Y(i) in Equation (2), we modify Equation (2) by normalization. The normalized SCC of each measurement can be obtained by the following.

(3) $$\begin{array}{c} \begin{array}{c} SCC_{m}^{*}=\frac{S_{m}}{S_{1}+S_{2}+\ldots +S_{M}}=\frac{\sum_{i=1}^{K}\left|\frac{X_{m}\left(i\right)-\bar{X_{m}}}{\sigma_{X_{m}}}\right|}{\sum_{m=1}^{M}\sum_{i=1}^{K}\left|\frac{X_{m}\left(i\right)-\bar{X_{m}}}{\sigma_{X_{m}}}\right|} \end{array} \end{array}$$

From Equation (3), we can conclude that the value of SCC is between zero and one; the larger the SCC, the higher the causality is. For example, when the value is zero, it means that the feature is not useful for predicting the target variable. We can see that the SCC given by Equation (3) omits the calculation process for the prediction Y(i).

**Figure 2:**
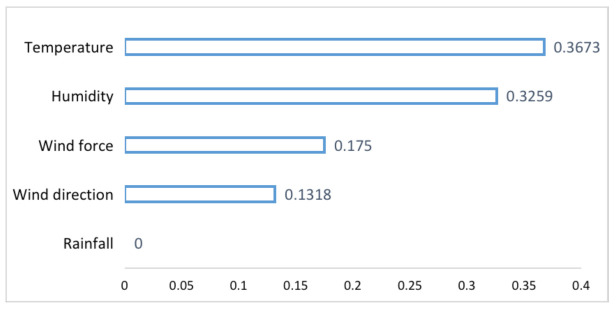
SCC between different measurements and predictions.

We give the following examples to illustrate the SCC obtained by Equation (3). Meteorological data are used, including temperature, wind direction, wind force, rainfall, and humidity, which are used to predict the future temperature. We have five measurements, so according to Equation (3), *M* is five. We set K=24, then $SCC_{m}$ can be obtained. X is set to five meteorological elements separately, and Y is the future temperature to be predicted. The result is shown in Table 1. To clearly illustrate the difference of $SCC_{m}$, we visualize them as Figure 2.

It can be seen from Table 1 and Figure 2 that the causality between historical temperature data and their future prediction is the largest, which is 0.3673. Next is humidity. We get the SSC as 0.3259 between historical humidity and future temperature. Compared with them, the causal relationship between wind force and wind direction with the future temperature is smaller. The data can also reflect no causal relationship between rainfall and temperature, for which we obtain a zero SSC.

Further, we can get the following conclusions. If all the data are used for training, rainfall data can only cause the network to reduce the training’s convergence and the temperature prediction performance. Therefore, rainfall data must be eliminated and cannot be used as the input for network training and prediction. Regarding wind force and wind direction data, because of their low causality, even as the network’s input data, the performance improvement of the prediction results is limited. It will increase the training time of the network. On the contrary, the humidity data have a high causal correlation with the future temperature. Therefore, using the historical temperature data and humidity data to predict the future temperature may achieve better performance than just using temperature data. The experiments in Section 4 will verify the above points.

### 3.2. Bayesian LSTM as the Sub-Predictor

**Figure 3:**
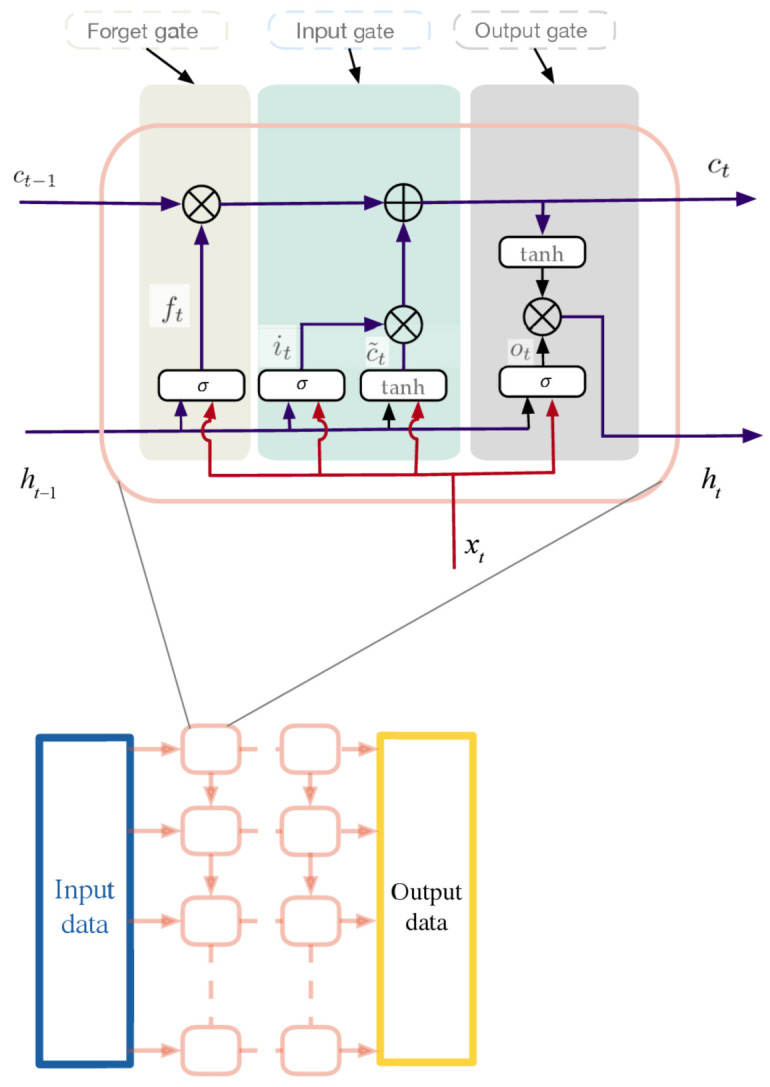
LSTM cell and its networks.

The LSTM cell is used in this paper, which is composed of three gating units, i.e., input gate, forget gate, and output gate. The calculation process is the following:

(4) $$\begin{array}{c} \begin{array}{c} \begin{array}{cc} f_{t} & =\sigma \left(W_{fx}x_{t}+W_{fh}h_{t-1}+b_{f}\right)\\ i_{t} & =\sigma \left(W_{ix}x_{t}+W_{ih}h_{t-1}+b_{i}\right)\\ {\bar{c}}_{t} & =\tanh\left(W_{cx}x_{t}+W_{ch}h_{t-1}+b_{c}\right)\\ c_{t} & =f_{t}\cdot c_{t-1}+i_{t}\cdot {\bar{c}}_{t}\\ o_{t} & =\sigma \left(W_{ox}x_{t}+W_{oh}h_{t-1}+b_{o}\right)\\ h_{t} & =o_{t}\cdot \tanh\left(c_{t}\right) \end{array} \end{array} \end{array}$$

where *t* is the current moment to predict, $w=[W_{fx},\;W_{fh},\;W_{ix},\;W_{cx},\;W_{ox},\;W_{oh}]$ are the weights, and $b=[b_{f},\;b_{i},\;b_{o}]$ are the biases. $c_{t}$ is the hidden state, and $h_{t}$ is the output of the LSTM cell. The cells can be placed as several layers with different input and output cells depending on the number of input and output steps of the prediction. The structure of the network is shown in Figure 3. The input data *x* are the given data used to predict the future trend, where x=[X(1),X(2),……,X(I)] are the input data at each moment with the number of data *I*, and $x_{t}=\left[X_{t}\left(1\right),\;X_{t}\left(2\right),\;\ldots \ldots ,\;X_{t}\left(I\right)\right]$ are the input data at the current moment *t*. The output of the last layer can be set as the output of the LSTM network, named as *y*. For the training process, we have y=[Y(1),Y(2),……,Y(J)], and at the current moment *t*, we have $y_{t}=\left[Y_{t}\left(1\right),\;Y_{t}\left(2\right),\;\ldots \ldots ,\;Y_{t}\left(J\right)\right]$.

**Figure 4:**
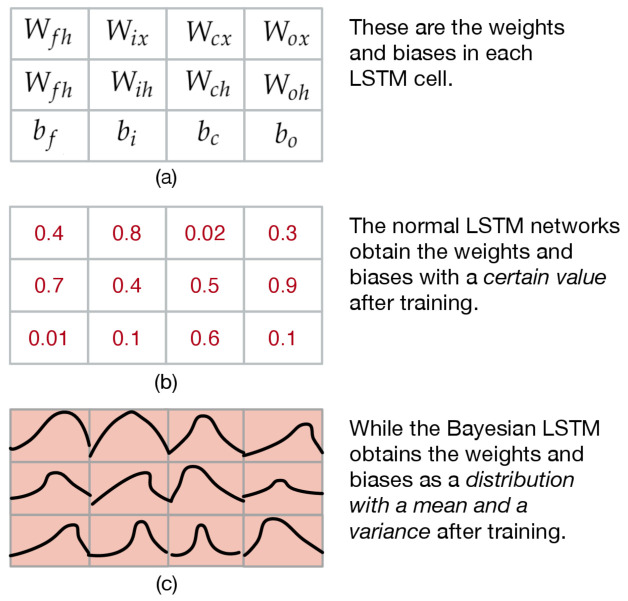
The difference between the normal LSTM network and the Bayesian LSTM network. (**a**) The parameters in the LSTM; (**b**) the example of the parameters in the normal LSTM; (**c**) the example of the parameters in the Bayesian LSTM.

In the normal LSTM network, the parameters, including all the weights and biases, are constants. The Bayesian LSTM can get the weight and bias as a random distribution, not a certain value. Each parameter obtained by the Bayesian LSTM network training is the mean and variance according to the distribution of the weights and biases. The difference between the normal LSTM network and the Bayesian LSTM network is shown in Figure 4.

The LSTM neural network can be seen as a probabilistic model P(y|x,θ): a probability given an input $x\;\in \;R^{p}$ to each possible output y∈Y, using the set of parameters θ including weights w and biases *b*, i.e., θ=[w,b]. We denote the training data *x* and *y* as *D*, i.e., D=[x,y].

Given the training data *D*, Bayesian inference can be used to calculate the posterior distribution of weights P(w|D) [62]. This distribution answers the predicted distribution of unknown data through the input data value: the predicted distribution of the input data *x* is given by $P\left(y\;|\;x\right)\;=\;E_{P(\theta \;|\;D)}\left[P\left(y\;|\;x,\;\theta \right)\right]$. Until now, it is still difficult to find P(w|D). The variational approximation to the Bayesian posterior distribution on the weights is a feasible method. Variational learning finds the parameters (μ,σ) of a distribution on the weights q(θ|μ,σ) that minimizes the Kullback–Leibler (KL) divergence [63] with the true Bayesian posterior on the weights:

(5) $$\begin{array}{c} \begin{array}{c} {\left(\mu ,\;\sigma \right)}^{*}=arg\;\underset{\mu ,\;\sigma }{min}\;KL\left[q\left(\theta |\mu ,\;\sigma \right)||P\left(\theta |D\right)\right] \end{array} \end{array}$$

According to the Bayesian theory,

(6) $$\begin{array}{c} \begin{array}{c} P\left(\theta |D\right)=\frac{P(D|\theta )P(\theta )}{P(D)} \end{array} \end{array}$$

and the definition of the Kullback–Leibler (KL) divergence, Equation (5) can be transformed to:

(7) $$\begin{array}{c} \begin{array}{c} {\left(\mu ,\;\sigma \right)}^{*}=arg\;\underset{\mu ,\;\sigma }{min}\int q\left(\theta |\mu ,\;\sigma \right)\log\frac{q(\theta |\mu ,\;\sigma )}{P(\theta )P(D|\theta )}d\theta \end{array} \end{array}$$

Note that we discarded P(D) because it does not affect the optimized parameter solution. Then, the cost function is set as:

(8) $$\begin{array}{c} \begin{array}{c} Loss=\int q\left(\theta |\mu ,\;\sigma \right)\log\frac{q(\theta |\mu ,\;\sigma )}{P(\theta )P(D|\theta )}d\theta \end{array} \end{array}$$

To keep the variance non-negative, we set it as σ=log(1+exp(ρ)). Set ε as zero mean Gaussian white noise, i.e., ε∼N(0,1). Then, we have θ=μ+log(1+exp(ρ))⊗ε, where ⊗ is point-wise multiplication. Further, we can note that q(θ|μ,ρ)dθ=q(ε)dε, then the derivative of Equation (8) can be calculated as the following:

(9) $$\begin{array}{c} \begin{array}{c} \frac{\partial }{\partial \mu }Loss=\frac{\partial }{\partial \mu }\int q\left(\theta |\mu ,\;\rho \right)\log\frac{q(\theta |\mu ,\;\rho )}{P(\theta )P(D|\theta )}d\theta \end{array} \end{array}$$

(10) $$\begin{array}{c} \begin{array}{c} \frac{\partial }{\partial \rho }Loss=\frac{\partial }{\partial \rho }\int q\left(\theta |\mu ,\;\rho \right)\log\frac{q(\theta |\mu ,\;\rho )}{P(\theta )P(D|\theta )}d\theta \end{array} \end{array}$$

Then, as for Equation (9), we have:

(11) $$\begin{array}{c} \begin{array}{c} \begin{array}{c} \frac{\partial }{\partial \mu }Loss=\frac{\partial }{\partial \mu }\int q\left(\theta |\mu ,\;\rho \right)\log\frac{q(\theta |\mu ,\;\rho )}{P(\theta )P(D|\theta )}d\theta \\ \;\;\;\;\;\;\;\;\;\;\;=\frac{\partial }{\partial \mu }\int \log\frac{q(\theta |\mu ,\;\rho )}{P(\theta )P(D|\theta )}\;q\left(\theta |\mu ,\;\rho \right)d\theta \\ \;\;\;\;\;\;\;\;\;\;\;=\frac{\partial }{\partial \mu }\int \log\frac{q(\theta |\mu ,\;\rho )}{P(\theta )P(D|\theta )}\;q\left(\varepsilon \right)d\varepsilon \\ \;\;\;\;\;\;\;\;\;\;\;=\frac{\partial }{\partial \mu }\log\frac{q(\theta |\mu ,\;\rho )}{P(\theta )P(D|\theta )}\int \;q\left(\varepsilon \right)d\varepsilon \\ \;\;\;\;\;\;\;\;\;\;\;=\frac{\partial }{\partial \mu }\log\frac{q(\theta |\mu ,\;\rho )}{P(\theta )P(D|\theta )} \end{array} \end{array} \end{array}$$

Similarly, Equation (10) can be derived further as the following:

(12) $$\begin{array}{c} \begin{array}{c} \frac{\partial }{\partial \rho }Loss=\frac{\partial }{\partial \rho }\log\frac{q(\theta |\mu ,\;\rho )}{P(\theta )P(D|\theta )} \end{array} \end{array}$$

Denote that:

$$\begin{array}{c} Loss=\log\frac{q(\theta |\mu ,\;\rho )}{P(\theta )P(D|\theta )}=\log q\left(\theta |\mu ,\;\rho \right)-\log P\left(\theta \right)-\log P\left(D|\theta \right) \end{array}$$

then we have:

(13) $$\begin{array}{c} \begin{array}{c} \begin{array}{c} \frac{\partial }{\partial \mu }Loss=\frac{\partial Loss}{\partial \theta }\frac{\partial \theta }{\partial \mu }+\frac{\partial Loss}{\partial \mu }\\ \;\;\;\;\;\;\;\;\;\;\;\;=\frac{\partial Loss}{\partial \theta }+\frac{\partial Loss}{\partial \mu } \end{array} \end{array} \end{array}$$

(14) $$\begin{array}{c} \begin{array}{c} \begin{array}{c} \frac{\partial }{\partial \rho }Loss=\frac{\partial Loss}{\partial \theta }\frac{\partial \theta }{\partial \rho }+\frac{\partial Loss}{\partial \rho }\\ \;\;\;\;\;\;\;\;\;\;\;\;=\frac{\partial Loss}{\partial \theta }\frac{\varepsilon }{1+exp(-\rho )}+\frac{\partial Loss}{\partial \rho } \end{array} \end{array} \end{array}$$

Please note that the standard deviations of the $\frac{\partial Loss}{\partial \theta }$ term of the mean and the gradient are shared, and it happens to be the gradient found by the backpropagation algorithm on the normal LSTM network. Therefore, to learn the mean and standard deviation, we can calculate the gradient by backpropagation and then scale and translate it. We summarize the optimization process as seven steps in Table 2.

### 3.3. Model Framework

**Figure 5:**
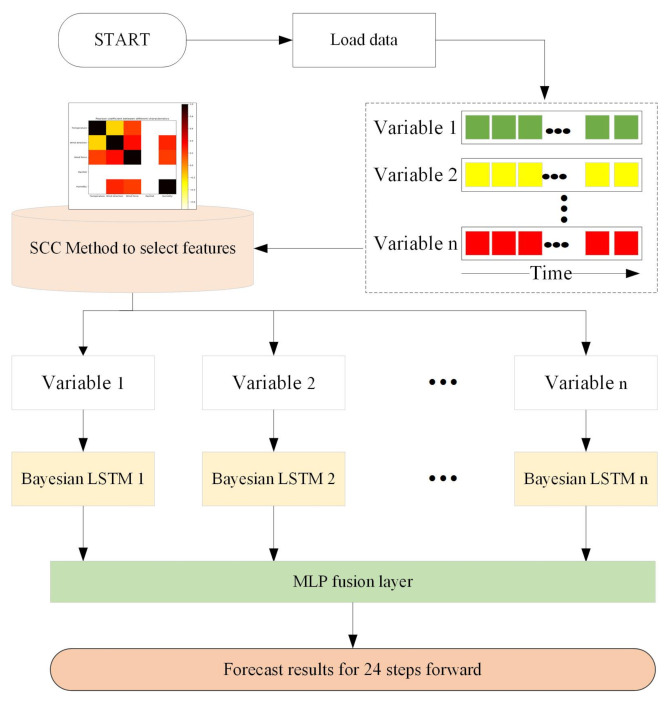
Model framework.

We propose a distributed prediction model combining SCC and a deep learning network for the prediction problem. The proposed model framework is shown in Figure 5, and the model consists of three main components: selection nodes, sub-predictors, and fusion nodes.

The selection node calculates the series causality of the data source and selects the variables related to the target data as the network input. For each selected input variable, a Bayesian LSTM sub-predictor is designed. Finally, we use the fusion node to fuse the prediction results of multiple sub-predictors. An artificial neural network MLP is used in the fusion node. MLP is a fully linked combination of artificially designed neurons, which applies a nonlinear activation function to model the relationship between the input and output.

## 4. Experiments

### 4.1. Dataset

Our experiments used the meteorological dataset in Shunyi District, Beijing, from 2017 to 2019. The data were measured hourly at meteorological station. The future temperature was chosen to be predicted to test the proposed model. The data set contained 1095 days for a total of 26,280 data samples to ensure sufficient training data. We selected the first 90% of the data for training and the remaining 10% for testing.

### 4.2. Experimental Setup

A PC with an Intel CORE CPU i5-4200U 1.60 GHz and 6 GB of memory was used for the experiments. In the experiments, the default parameters in Keras and Pytorch were used for deep neural network initialization. We used the ReLU as the activation function of the Bayesian LSTM layer and the linear activation function of the MLP layer.

We set up one Bayesian LSTM layer and one MLP layer, and each layer’s size was set to 24. The Adam algorithm was used for the supervised training, and the model was trained by mini-batch sampling. The model hyperparameters, such as learning and batch size, were obtained from experiments and are presented in Table 3.

The model’s performance was evaluated by the following four factors. The root-mean-squared error (RMSE):

(15) $$RMSE=\sqrt{\frac{1}{n}\sum_{i=1}^{n}{\left(y_{i}-{\hat{y}}_{i}\right)}^{2}}$$

where ${\hat{y}}_{i}$ is the prediction, $y_{i}$ is the ground truth, and n is the number of data.

The mean-squared error (MSE) can reflect the value of the loss function of network convergence and is defined as:

(16) $$\begin{array}{c} \begin{array}{c} MSE=\frac{1}{n}\sum_{i=1}^{n}{\left(y_{i}-{\hat{y}}_{i}\right)}^{2} \end{array} \end{array}$$

The mean absolute error (MAE) and Pearson correlation coefficient (R) between the prediction and reference were also explored in the experiments.

(17) $$\begin{array}{c} \begin{array}{c} MAE=\frac{1}{n}\sum_{i=1}^{n}\left|y_{i}-{\hat{y}}_{i}\right| \end{array} \end{array}$$

(18) $$\begin{array}{c} \begin{array}{c} R=\frac{\sum_{i=1}^{n}\left(y_{i}-{\bar{y}}_{i}\right)\left({\hat{y}}_{i}-{\bar{\hat{y}}}_{i}\right)}{\sqrt{\sum_{i=1}^{n}{\left(y_{i}-{\bar{y}}_{i}\right)}^{2}\sum_{i=1}^{n}{\left({\hat{y}}_{i}-{\bar{\hat{y}}}_{i}\right)}^{2}}} \end{array} \end{array}$$

### 4.3. Case 1

In this case, the Bayesian LSTM model’s performance is verified and causality evaluated by predicting the further temperature. We used the SCC to compare the correlation between time series variables and selected the temperature and humidity as the distributed deep model’s input data. We set the time step to 24 and got a total of 24 prediction steps. The blue and red lines present the ground truth of temperature and the model’s predictive results, respectively. The RMSE of the prediction is 3.203.

**Figure 6:**
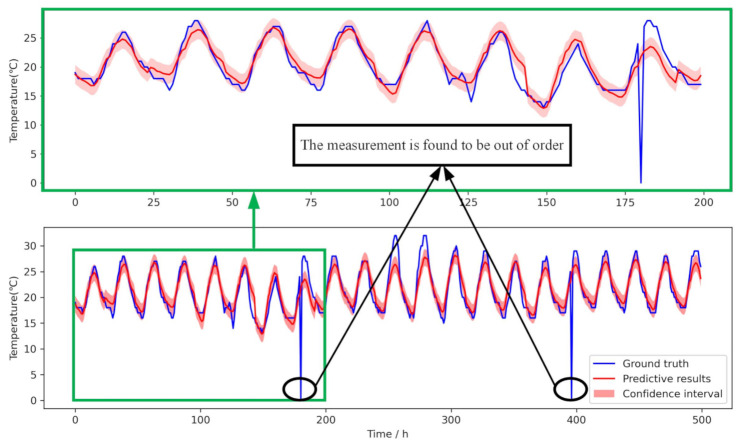
The prediction results of the temperature. The above picture is the prediction for the first 200 hours, which is a part of the bottom picture, in which we draw the results for about 21 days. We can see that in the bottom picture, the sensor is out of order with two hours, in which the sensor measurement data are zero. However, the prediction result effectively overcomes the sensor’s failure and gives a daily temperature trend consistent with historical data.

Figure 6 shows the comparison of the measurement data (the ground truth) and the 24 step forward prediction results. There is a light red band above and below the red line, which is the variance of the Bayesian network’s result. It can be seen that the predictive trend is close to the ground truth, and most of the forecast values are within the confidence interval.

From the actual measurement data, the prediction model’s input data caused by sensor failure give the wrong measurement value. We can see that in the bottom picture, the sensor is out of order in two hours, in which the sensor measurement data are zero. However, the prediction result effectively overcomes the sensor’s failure and gives a daily temperature trend consistent with historical data. However, the prediction result still maintains the correct trend, which effectively overcomes the sensor failure.

### 4.4. Case 2

In this case, we calculated the causality of the four meteorological factors in the data set and selected the best data for the network model. Because the SCC is zero between temperature and rainfall, we did not consider the rainfall data in the prediction.

The data set used to predict the temperature is four meteorological elements, i.e., historical temperature, humidity, wind force, and wind direction. We first considered two variables as the input of the network. We found that the predicted performance was different in different combinations. This performance was related to the SCC parameter. In another case, we increased the input signal to three or four. The results show that as the sensor input data increased, the prediction performance would not improve, but would decrease instead.

**Figure 7:**
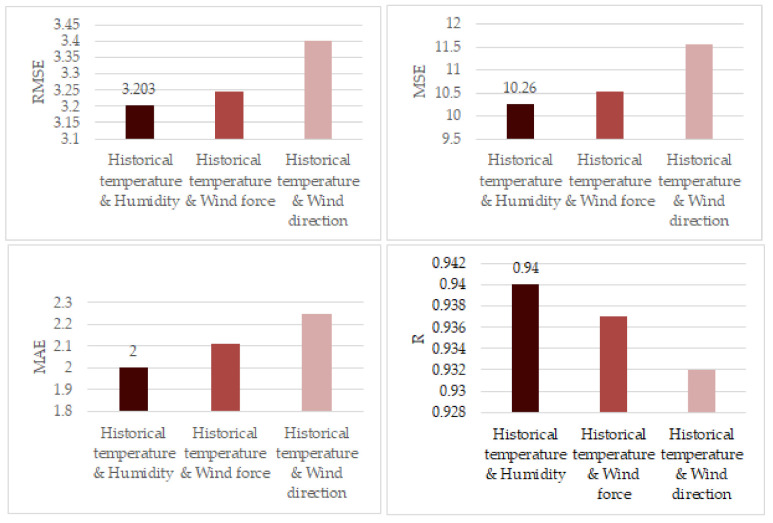
Comparison of prediction performance with two inputs. The input variables are historical temperature and humidity, historical temperature and wind force, and historical temperature and wind direction, respectively. We can find that when the inputs are the historical temperature and humidity, the least RMSE, MSE, and MAE and the largest R can be obtained.

Table 4 and Figure 7 show the comparison results with two inputs. It can be seen from Table 4 that when historical temperature and humidity are set as the input, the best prediction performance can be obtained, in which the RMSE, MSE, and MAE are 3.203, 10.260, and 2, respectively. Compared with other combinations of input, such as historical temperature and wind force and historical temperature and wind direction, the RMSE, MSE, and MAE decreased.

The larger the SCC, the more it shows that the data have more causality with respect to the target data. As shown in Table 1, the historical temperature data and humidity have the greatest correlation with the future temperature data. Therefore, using these two types of data, compared with historical temperature data as the input, we can significantly improve the prediction performance.

**Figure 8:**
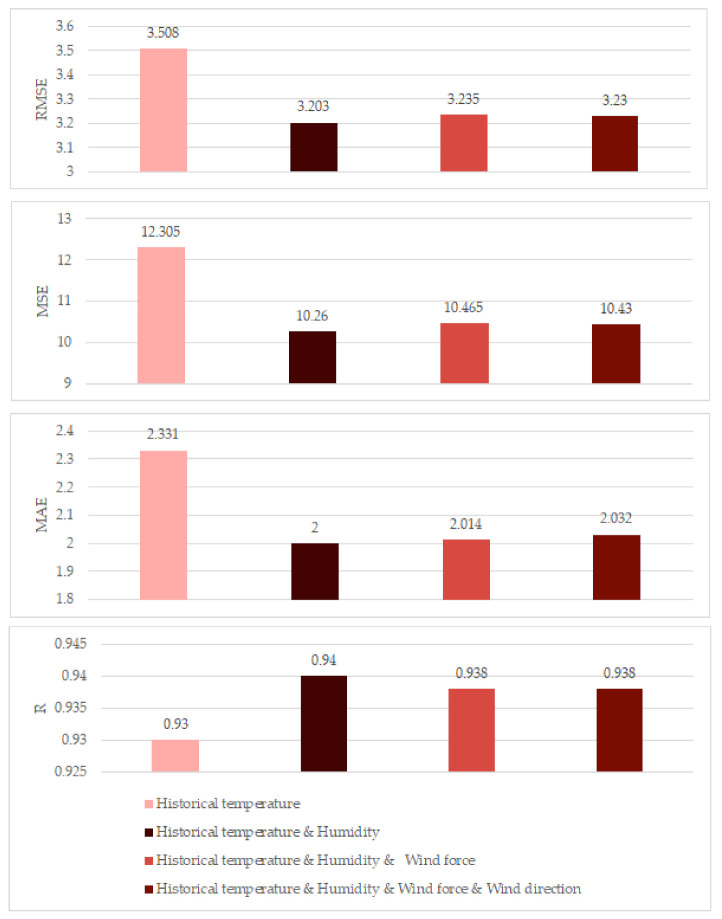
Comparison of prediction performance with multiple inputs. We can see that when two input variables are used, compared with one input variable, the RMSE, MSE, and MAE decrease and R increases, which shows that the performance is getting better. However, as the number of input variables increases, the performance becomes worse. For example, when the input variables are historical temperature, humidity, and wind force, the prediction performance worsens. Further, when we use the four input variables, the performance is the worst.

Then, we increased the input variables one-by-one, adding humidity, wind force, and wind direction, separately. The performance of different numbers of inputs are shown in Table 5 and Figure 8. We can see that when there was only historical temperature as the input data, the RMSE, MSE, and MAE were 3.508, 12.305, and 2.331, respectively. Then, when two inputs were used, that is together historical temperature with humidity, the minimum prediction RMSE was 3.203. In addition, the MAE, MSE, and R were the best also. However, when the input data increased and three input data were used, the RMSE increased to 3.235. When four input data were used, the RMSE further increased to 3.230. Therefore, we can conclude that the experiments show that more input data do not result in better prediction performance.

### 4.5. Case 3

**Figure 9:**
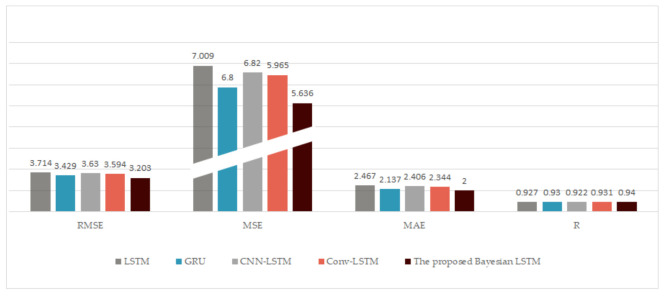
Comparison of the prediction performance with different sub-predictors. We can find that the proposed model with the Bayesian LSTM is the best, obtaining the least RMSE, of 2.374, MSE, and MAE and the largest R.

In this case, we compared other deep network models with the methods proposed in this paper. Among them, no baseline models included a feature selection process and used all features as the network input. As shown in Table 6 and Figure 9, the RMSEs of LSTM [27], GRU [28], CNN-LSTM [11], conv-LSTM [30], and the proposed Bayesian LSTM were 3.714, 3.429, 3.630, 3.594, and 3.203 and the MSEs were 13.797, 11.759, 13.174, 12.915, and 10.260, respectively. The MAEs were 2.467, 2.137, 2.406, 2.344, and 2.000, respectively. Compared with LSTM and GRU, the RMSE of the proposed Bayesian LSTM decreased by 13.76% and 6.59%, and the MSE decreased by 25.64% and 12.75%, while the MAE decreased by 18.93% and 6.41%, respectively. Compared with other hybrid models, such as CNN-LSTM and conv-LSTM, the results show that the Bayesian LSTM was the best, obtaining the minimum RMSE of 3.203 and the least MAE of 2.000. Therefore, the Bayesian LSTM can better fit the data and had the best prediction performance.

## 5. Conclusions

This article focuses on multivariate noisy measurement data modeling and prediction and proposes a distributed deep Bayesian LSTM prediction network based on causality entropy. The performance of the model was verified on real weather data sets.

In a multi-sensor system, the actual data set is usually non-linear and noisy. Therefore, analyzing the correlation between measurement from a multi-sensor system is very important for predicting. We developed the SCC to analyze the original multidimensional variables and then selected the most causal variable for the target variable. The SCC can reduce the total amount of data entered into the network, thereby reducing the computational burden of the network. It also reduces errors caused by unnecessary input.

As we all know, neural networks have a strong ability to fit nonlinearity. However, we found that the measurement data from the multi-sensor system have complex noise. We used the Bayesian LSTM to reduce the influence of noise on the neural network. The model was modeled by weight sampling, and then, the average was taken to obtain a more stable output.

In future research, we can consider other causality analysis methods. We will also replace the MLP with other fusion methods to reduce the network model’s parameters for the fusion results. The proposed approaches in the paper can combine other parameter estimation algorithms [32,64,65,66,67] to study the parameter identification problems of linear and nonlinear systems with different disturbances [68,69,70,71,72], and to build the soft sensor models and prediction models and can be applied to other fields [73,74,75,76,77] such as signal processing and process control systems.

## Figures and Tables

**Table 1:** The order of the SCC between variables to be predicted.

Measurement	Series Causality Coefficient (SCC)
Temperature	0.3673
Humidity	0.3259
Wind force	0.175
Wind direction	0.1318
Rainfall	0

**Table 2:** The optimization process for the Bayesian LSTM networks.

Step	Optimization Process
0	Set the scale parameter α as α∈(0,1).
1	Sample the random variable ε as ε∼N(0,1).
2	Set the initial value of the optimized parameters (μ,ρ).
3	Sample all the parameters as θ=μ+log(1+exp(ρ))⊗ε.
4	Set the cost function as Loss=logq(θ|μ,ρ)−logP(θ)+logP(D|θ).
5	Calculate the gradient by the mean with the training data *D* as $△\mu =\frac{\partial Loss}{\partial \theta }+\frac{\partial Loss}{\partial \mu }$.
6	Calculate the gradient by the standard deviation with the training data *D* as $△\rho =\frac{\partial Loss}{\partial \theta }\frac{\varepsilon }{1+exp(-\rho )}+\frac{\partial Loss}{\partial \rho }$.
7	Update the parameters (μ,ρ) as the following: μ←μ−α△μ,ρ←ρ−α△ρ.

**Table 3:** Hyperparameters for the experiments.

Layers	Design Details	Experiment Setup
Bayesian LSTM	Number of layers: 1 Number of neurons: 24 Sampling number: 4	Batch size: 30 Epochs: 100 Learning rate: 0.001
MLP	Number of layers: 1 Number of neurons: 24	

**Table 4:** Prediction performance with two inputs.

Input Data	RMSE	MSE	MAE	R
Historical temperature and humidity	3.203	10.260	2.000	0.940
Historical temperature and wind force	3.244	10.525	2.108	0.937
Historical temperature and wind direction	3.400	11.559	2.250	0.932

**Table 5:** Prediction performance with multiple inputs.

Input Data	RMSE	MSE	MAE	R
Historical temperature	3.508	12.305	2.331	0.930
Historical temperature and humidity	3.203	10.260	2.000	0.940
Historical temperature, humidity, and wind force	3.235	10.465	2.014	0.938
Historical temperature, humidity, wind force, and wind direction	3.230	10.430	2.032	0.938

**Table 6:** Prediction performance with different models.

Model	RMSE	MSE	MAE	R
LSTM [27]	3.714	13.797	2.467	0.927
GRU [28]	3.429	11.759	2.137	0.930
CNN-LSTM [11]	3.630	13.174	2.406	0.922
Conv-LSTM [30]	3.594	12.915	2.344	0.931
The proposed Bayesian LSTM	3.203	10.260	2.000	0.940

## Data Availability

The data presented in this study are available on request from the corresponding author.
